# Trajectories of physical functioning among older adults in the US by race, ethnicity and nativity: Examining the role of working conditions

**DOI:** 10.1371/journal.pone.0247804

**Published:** 2021-03-17

**Authors:** Anne R. Pebley, Noreen Goldman, Theresa Andrasfay, Boriana Pratt

**Affiliations:** 1 California Center for Population Research, Fielding School of Public Health, University of California, Los Angeles, California, United States of America; 2 Office of Population Research, Woodrow Wilson School of Public and International Affairs, Princeton University, Princeton, New Jersey, United States of America; 3 Leonard Davis School of Gerontology, University of Southern California, Los Angeles, California, United States of America; 4 Office of Population Research, Princeton University, Princeton, New Jersey, United States of America; Graduate School of Public Health and Health Policy, City University of New York, UNITED STATES

## Abstract

Latinos in the US live significantly longer than non-Latino whites, but spend more years disabled. Differentials in socioeconomic status account for part, but not all, of the difference in older age disability between Latinos and whites. We hypothesize that a factor often ignored in the literature—the fact that Latinos, on average, have more physically strenuous jobs than non-Latino whites—contributes to the higher Latino risk of functional limitations at older ages. We use longitudinal data from the 1998–2014 Health and Retirement Study (HRS) comprising 17,297 respondents. Compared to US-born whites, Latinos, especially Latino immigrants, report substantially higher levels of physical effort at work. Latino-black differences are much smaller than Latino-white differences. As hypothesized, physical work effort is strongly related to functional limitations. However, differentials in physical work effort for Latinos and whites in their fifties and early sixties are weakly related to Latino-white differentials in FL at later ages.

## Introduction

Although Latinos in the US live significantly longer than non-Latino whites [1], they spend more years disabled. In 1989–2006, for example, life expectancy for foreign-born Latina women was 2.7 years longer than for non-Latina white (hereafter “white”) women, but Latinas spent five more years with a disability [2]. Disability creates hardship for individuals and families. It also reduces individuals’ ability to work, thus potentially exacerbating social inequality. Latinos are also less likely than whites to have health insurance and the financial resources to cope with disability and its consequences. Understanding sources of racial and ethnic inequality in disability can help identify means to reduce disability rates in higher risk groups and the general population.

Why would Latinos in the US be more likely to have disability and functional limitations (FL) than whites, despite having better health on other indicators [1]? FL and disability are strongly associated with socioeconomic status (SES, i.e., education, income, wealth, occupational rank, and parental SES) as is race/ethnicity, but SES differentials alone cannot account for racial/ethnic differences in FL and disability [3–5]. Previous research suggests that other mechanisms are also involved, including racial/ethnic inequalities in: access to health care, early life conditions, exposure to environmental hazards, personal health behaviors, and stress [6–8]. However, most research on the determinants of disability and FL ignores another potentially important mechanism: substantial differences in the work environment for Latinos (especially foreign-born), whites, and workers from other ethnic groups, and the cumulative effects of differential physical and psychological work conditions on older age FL and disability [9–11]. Latinos (especially immigrants) are more likely than whites to work in manual jobs requiring heavy physical, ergonomically challenging, and hazardous work [12–18]. Extensive research on occupational health has shown a strong relationship between work conditions and health outcomes [19]. Thus, differentials in work conditions are a plausible explanation for observed differentials in FL between the two groups.

In this paper, we examine racial, ethnic, and nativity (hereafter REN) differentials in limitations in physical functioning (e.g., walking, lifting), rather than in disability per se. In the disablement process, FL are necessary but not sufficient, precursors for developing a disability. Disability itself depends on the demands of, and supports available in, a person’s environment [20, 21]. In terms of differentials in the work environment by REN, we focus on physical work conditions because they are especially likely to be associated with musculoskeletal and other FL-related conditions. However, psychosocial work conditions may also negatively affect physical functioning. Our central hypothesis is that REN differences in physical work conditions contribute to REN disparities in physical functional limitations at older ages. To determine whether there is evidence consistent with this hypothesis, we use longitudinal data from the US Health and Retirement Study (HRS). Our analysis examines three central questions. First, to what degree are Latinos disadvantaged relative to whites and blacks in both FL trajectories and physical work conditions in the HRS sample, as has been shown in the literature with other data? Second, is heavier physical work during the 50s and early 60s associated with more FL at older ages? Third, do REN differences in physical work conditions account for a significant proportion of the REN disparities in FL at older ages?

We focus on Latino-white and nativity differentials, but include non-Latino blacks (hereafter, African Americans or blacks) who also experience higher levels of functional limitations (FL) than whites, as an alternative comparison group. Our results are stratified by gender because of large gender differences in occupational and disability patterns.

To our knowledge, this paper is the first to investigate whether racial/ethnic, nativity, and gender differentials in strenuous physical work are associated with subsequent trajectories of older age functional limitations in a nationally representative sample of the US population. As noted below, most previous research on this topic has been conducted in European populations, which have substantial cultural, racial/ethnic, and health differences from the US. Our paper extends the literature on social inequalities in health by investigating a potentially important, but frequently ignored, mechanism through which these inequalities may occur: social differentials in work conditions. Despite the extensive occupational health and safety literature [11, 19] research on the relationship between differential work conditions by REN and SES and long term health is limited [14, 22].

## Background

### Racial, ethnic and nativity differences in functional limitations

Previous studies have examined *disability* by REN in the US, but few have investigated differences in *functional limitations* (FL). Those that do find consistently higher limitations among blacks than whites; some also find that Latinos (or, in some cases, Mexican-origin individuals) have more limitations than whites, but the Latino-white differential is not consistent across studies [6, 23–25]. The study most relevant to our analysis is Haas and Rohlfsen [6], which analyzes longitudinal 1992–2004 US Health and Retirement Study (HRS) data. The authors conclude that both Latinos and blacks have substantially higher numbers of limitations than whites, although Latino and white trajectories of FL converge at older ages. However, they do not examine the potential role played by occupational segregation and associated physical work conditions on FL. Nor do they look at nativity differentials which may be especially important for the Latino population.

Functional limitations have been shown to be less predictive of mortality for blacks and Latinos than for whites, suggesting that although these groups experience more FL, this higher FL prevalence may partly result from wear and tear and injuries that do not necessarily lead to higher mortality [26].

### Work conditions and functional limitations at older ages

Work is highly stratified by REN and gender [12, 27]. In 2006–2010, for example, 70% of Latino men held manual labor occupations compared to 58% of black men and 46% of white men. For women, comparable figures were 43% for Latinas, 35% for black women, and 23% for white women [28]. There is also a large gap between US-born and foreign-born Latinos and, among immigrants, by documentation status [15, 18, 29]. Latinos, particularly undocumented immigrants, are more likely to hold hazardous and physically strenuous jobs than whites [18, 30]. Undocumented immigrants are also more likely to work in informal and poorly regulated jobs [18, 31–33].

Since the 1800s, research has demonstrated that physical work conditions affect health through exposure to hazardous substances, injury, and long-term wear and tear as well as higher disease prevalence [19]. Blue-collar jobs often involve exposure to physical hazards [11] including moving heavy loads, harmful positions (e.g., bending, crouching), repetitive motion, heights, hazardous equipment, and limited physical movement. Although regular physical activity is important for health, the strenuous physical activity in many blue-collar jobs has been shown to be harmful [34, 35]. Studies, mostly from Europe, have linked strenuous physical work conditions to FL and poor health in later life [36–40]. To the best of our knowledge, no studies have examined these linkages among race/ethnicity, strenuous work conditions and FL in the US.

### Other factors: Early life conditions, socioeconomic and marital status, and health

Physical work conditions may be associated with subsequent functional limitations for reasons aside from a direct causal link. These reasons include: health conditions and behaviors, early life conditions and educational attainment, adult socioeconomic status (SES), and marital status. Thus, our analysis includes these factors as control variables.

Health behaviors and conditions, like smoking and obesity, are strongly associated with functional limitations and vary considerably by race, ethnicity, and nativity [41–43]. Not surprisingly, health conditions at middle and older ages which are linked to those behaviors, such as diabetes and heart disease, also vary by REN and can have a substantial impact on the risk of developing FL [44–47].

The same life circumstances and social structure that can lead Latinos and African Americans to work more often than whites in physically strenuous jobs may also, independently, affect their likelihood of experiencing late life FL: lower educational attainment, poorer early life conditions, parental occupations, and discrimination and structural racism [48–53]. An extensive literature suggests that an individual’s occupation and work conditions are determined primarily by parental social status, childhood disadvantage, and educational attainment [54–58]. Intergenerational social mobility in the US is relatively low and childhood disadvantage is strongly associated with poor educational and occupational attainment as well as income and wealth in adulthood [59]. Children in low-income families are also more likely to have poor health, including poorer physical and cognitive development, which may directly affect health outcomes in later adulthood, including FL [60, 61].

Socioeconomic status (SES) in adulthood is also strongly linked with race/ethnicity and nativity, and may account for a substantial part of the REN differentials in FL. SES is strongly associated with virtually all aspects of health—including older age FL [3, 62–64]. Many studies investigating the SES and FL relationship are cross-sectional and focus exclusively on educational attainment and *current* income and wealth. Contemporaneous measurement makes it impossible to identify the direction of association, since FL may also lead to lower income and wealth, and, depending on age of onset, lower educational attainment.

Few studies have investigated the role of SES in affecting FL at older ages. Those that do conclude that controlling for SES substantially reduces the differential risk of FL and/or disability between Latinos and whites [6, 62]. Haas and Rohlfsen [6] include both adult and childhood SES variables in their models. They find that inclusion of childhood disadvantage substantially reduces the Latino-white differential, but that the effects of childhood disadvantage are largely due to its correlation with adult SES [6].

Previous research also shows that marital status is associated with older adult health outcomes and with socioeconomic status [65]. Marital status varies considerably among racial and ethnic groups in the US, with whites more likely to be married and blacks less likely to be married, compared to Latinos [66].

### Data

We use data from the Health and Retirement Study (HRS), a US national longitudinal survey of individuals over 50 years of age, interviewed every two years since 1992 –initially in-person and subsequently by phone. HRS is the model for a series of national longitudinal surveys on aging in Europe, Asia, Latin America, and Africa [67]. It is based on a multi-stage clustered probability sample, with an oversample of blacks and Latinos. Respondents aged 51–61 were sampled in 1992 and cohorts aged 51–66 have been added every six years since then [68]. In sampled households, one respondent is picked at random from residents older than 50; if the respondent has a spouse, (s)he is also included in the sample, regardless of age. We use the HRS because, unlike other large, nationally-representative longitudinal surveys in the US, it focuses on a sample of older adults; collects well-tested measures of physical functioning, respondents’ physical work conditions, and socioeconomic status; and oversamples the Latino population.

Our data sets were constructed from the 2014 HRS tracker file and the RAND HRS files through 2014 (version 2). These files are public-use deidentified survey data collected and distributed by the University of Michigan’s Survey Research Center under approval by the Institutional Review Board (IRB) at the University of Michigan. This research was reviewed, classified as “exempt,” and approved by the IRBs at the University of California, Los Angeles and Princeton University.

We use data from waves (interviews) between 1998 and 2014, including proxy responses. Earlier waves are excluded because of variations in samples and questionnaires. Of the 176,277 person-waves in the total HRS sample for 1998–2014, we excluded 3.2% who were respondents (primarily spouses) younger than 50 and 2.5% who reported a race/ethnicity other than Latino, black or white. We also excluded 3.4% of observations who were spouses of the primary respondents in second or higher order marriages, thereby eliminating multiple spouses with the same household number. Finally, we excluded 0.85% of observations that were missing a response to at least one of the functional limitation questions (described below).

Given our goals, the sample was then restricted to respondents for whom physical work effort data are available. HRS data on employment and work effort are available primarily for jobs held during the survey years (1992 to 2014). For this reason, our sample includes only respondents who were working (employed for pay) at or close to their first interview in the observation period (i.e., 1998 to 2014) and who provided the information on work effort. The sample comprises 94,975 observations of 17,297 respondents (8,226 men and 9,071 women), for an average of 5.49 observations per person. Of the potential 103,498 observations that could have been included for these respondents, 8,523 observations (8.2%) were lost-to-follow-up (i.e., the respondent was alive but did not participate in the interview for a specific wave) and are therefore excluded from the analysis in the wave that is missing; all other observations for these respondents through 2014 are included. Most (13,627) of the 17,297 respondents in the analytic sample had information on physical work effort in 1998 or at their first interview post-1998. However, because not all employed respondents reported work effort at these times, this total also includes (a) 2,101 cases that reported work effort in a later (but not the first) interview in the observation period and (b) 1,569 cases that reported work effort before 1998 but not during the observation period. For cases in which work effort was first reported in a later interview in the observation period, we began observation of the FL trajectory with the wave in which work effort was reported. On average, respondents in the employed sample were 58 years old at the time they answered these questions.

Note that HRS contains no information on work conditions associated with unpaid housework or caregiving, which may also be physically demanding and vary substantially by gender and REN. Of necessity, we classify homemakers and individuals providing care for family members without pay as “not working,” along with those who are not working for pay for other reasons (e.g., unemployed, out of the labor force, retired).

### Measures

#### Outcome

The outcome variable is a measure of physical functioning. At each wave, respondents reported whether they could do specific tasks: walking several blocks; walking one block; sitting for two hours; getting up from a chair; climbing several flights of stairs without resting; climbing one flight of stairs without resting; stooping, kneeling or crouching; extending arms above shoulders; pushing or pulling large objects; lifting or carrying at least 10 pounds; and picking up a dime. Respondents reporting difficulty or inability with, or not performing, the task were considered to have the limitation, a strategy previously used with HRS and other survey data [69]. The questions were designed to exclude limitations lasting less than three months. Our outcome variable is the number of FL reported by the respondent from this list, summed to create a measure that ranges between 0 and 11.

#### Exposure

We consider two self-reported work effort measures likely to affect physical limitations: physical effort and heavy lifting. HRS asked employed respondents (1) whether their current “job requires lots of physical effort” and (2) whether their current “job requires lifting heavy loads,” and respondents indicated whether this is “true all or almost all of the time, most of the time, some of the time, or none or almost none of the time.” Both variables are dichotomized to distinguish most, almost all or all of the time from the other categories. We refer to these two variables collectively as “work effort” and to the individual variables as “heavy physical effort” and “heavy lifting.” As noted above and described below, we measure these variables at one point in time—at or close to the first interview in the observation period—and consider their effects on the subsequent trajectory of physical functioning for each respondent.

HRS occupational history data are limited primarily to jobs held during the survey years (1992 to 2014). Although some information on a maximum of two earlier jobs is available from respondents’ first interview (e.g., the most recent job—prior to the current or last job—with duration of at least five years), information about the level of physical effort is asked only for the current job at first interview plus current jobs at all subsequent interviews.

Thus, we restrict the job effort variables to a single point in time at or close to the first interview in the observation period. This provides a measure for respondents in their 50s or early 60s, when they are likely to be working in an occupation with a similar level of work effort as the main occupation they had held in their prime working years. Empirical evidence suggests that it is common for individual workers to have the same types of occupations over their prime working years—e.g., ages 30 to 60 –although in recent years, there has been some increase in lifetime career mobility [70]. Undoubtedly, as workers in jobs with high physical effort approach retirement, some may move into less physically demanding jobs within their occupational category, but other workers (e.g., undocumented immigrants, those with multiple dependents) may not have the choice. To the extent that HRS respondents moved to less strenuous jobs over their lifetimes, our analysis would provide a conservative test of our hypothesis. HRS data reveal that many older workers do have jobs requiring strenuous physical work. For example, close to 40 percent of our analytic sample reported that their work involved heavy physical effort.

#### Covariates

*Race*, *ethnicity*, *and nativity*. Our primary covariate of interest is race/ethnicity and nativity (REN). REN is coded into six groups, implicitly incorporating their interaction: Latino US-born, Latino foreign-born, black US-born, black foreign-born, white US-born (the reference group), and white foreign-born. Although self-classifications of race, ethnicity, and nativity can change over time, we used the time invariant variable provided by the HRS Tracking File.

*Age and wave*. Because functional limitations develop over time and during the aging process, the respondent’s age is a key variable throughout the analysis. Age at the time of each wave is treated as continuous and centered on 60 years. We also include the HRS wave number, from 4 (1998) to 12 (2014), as a period effect.

*Early life socioeconomic status (SES)*. We include eight early-life variables including family financial status from birth to age 16 (coded as fairly well off, about average, and poor); father’s employment status prior to age 16 (coded as father had periods of unemployment of at least three months, father did not have these periods, and father did not live with child); mother’s education in years; father’s education in years; whether the respondent lived in a rural area most of the time; health status prior to age 16 (coded as poor or fair vs. good, very good or excellent); height at first interview (in meters), which provides an indirect measure of childhood nutritional status and disease [71]; and completed years of schooling at first interview. All early-life variables were treated as time-invariant.

*Adult SES*. We include three adult SES-related variables. HRS collects detailed information at each interview regarding the respondents’ and spouses’/partners’ incomes (e.g., salaries and wages, business income and losses, investment income, income from transfers and public program receipts, etc.) for the calendar year prior to interview and wealth or assets (e.g., bank accounts, investments, home value, value of other assets etc.) at the time of the interview. The data files provide variables for income and wealth of the couple, which we refer to as household income and household wealth in the analysis. These income and wealth variables are time-varying and are coded into quartiles of real 1992 dollars (based on the full sample of HRS respondents). We also include whether the respondent is currently married as a binary time-varying measure.

*Adult health*. The models include three time-varying measures of adult health. The first is whether or not the respondent ever smoked. The second is the body mass index (BMI) in the three conventional obesity classes–(30–35), (35–40), and (40–45)–with non-obese as the reference category. We also include whether or not a doctor ever told the respondent that (s)he has diabetes or high blood sugar.

### Analytic strategy

The first goal of the analysis is to assess the level of REN differences by gender in both the outcome and exposure variables. We examine trajectories of physical functional limitations by age by REN and gender for the entire HRS sample to provide a picture of the size of REN and gender differentials at older ages in the United States. As described above, the nature of HRS data limit our analytic sample to respondents employed at the time of initial observation. If workers in jobs requiring heavy work effort are more likely to develop functional limitations, they may retire early, go on disability, or be unable to work and be excluded from the analytic sample. For this reason, we also investigate whether there are substantial differences in FL trajectories between the entire HRS sample and our analytic sample. The analysis is presented in S1 Appendix and summarized in the results section. We also describe racial and ethnic differentials in reports of heavy physical effort across occupations for our analytic sample.

The main part of the analysis addresses the other goals described in the introduction: (a) investigating the association between work effort and functional limitations and (b) whether REN differentials in physical work effort can account for REN differentials in older age functional limitations. Because the outcome is a count (the number of FL), we use Poisson regression, specifically a mixed effects Poisson regression model that includes a normally distributed random intercept to account for multiple observations per respondent. Poisson models typically provide a poor fit to such data because of overdispersion—i.e., variance in the observed data that greatly exceeds the mean, violating the assumption of equal mean and variance in a Poisson distribution. This situation arises because many respondents have no FL, resulting in a much larger proportion of zero values than in a Poisson distribution. The problem of excess zeroes has frequently been addressed with a two-part model (e.g., a zero-inflated Poisson model that separately models the probability of having zero limitations; see [72]). However, in this analysis the overdispersion is reduced sufficiently by inclusion of a random effect for individuals, thereby avoiding the complications of a two-equation model. In particular, a Poisson model with a random intercept provides a much closer fit to the observed data than a simple Poisson model [73]. For example, 36% of observations in our sample have zero limitations. A standard Poisson model including only age as an explanatory variable predicts only 12% with zero limitations whereas the corresponding random intercept Poisson model predicts 32%.

To investigate the association between work effort and functional limitations, we estimate two nested random intercept Poisson models, which we refer to as “trajectory models” since they examine person-level trajectories in functional limitations: **Model 1a**, which includes age, marital status, wave, adult health variables, and work effort variables; and **Model 1b**, which adds early life variables and measures of adult SES. Both models include linear and quadratic terms for age to capture the nonlinear increase in FL at older ages. They also include interactions between REN and age to allow for differential rates of increase of FL with age across groups (interactions with the quadratic age term were not significant in preliminary analyses and were thus not included). Exploratory analysis indicated that the association between work effort and limitations did not differ by REN so these interaction terms were not included. We use Models 1a and 1b to obtain insight into the strength of the relationship between work effort and FL by comparing the work effort coefficients to the corresponding estimates for the three adult health status variables described earlier that are known to have important effects on FL.

To determine whether REN differentials in physical work effort can account for REN differentials in FL, we estimate variations of the trajectory models above to examine the difference in predicted counts of FL by REN associated with the inclusion of work effort. **Model 2a** includes age (linear and quadratic terms), marital status, and wave. **Model 2b** additionally includes work effort variables. **Model 2c** includes all predictor variables with the exception of work effort variables; this corresponds to Model 1b without work effort variables.

We present these results as predicted counts of FL because the interaction term between REN and age in the trajectory models makes it difficult to discern the magnitude of REN differentials from the estimated coefficients. Predictions are essentially simulations of the outcome based on a particular set of inputs. They were calculated by assigning all individuals the same REN, setting age to a specific value, and keeping all other covariates at their observed values. Since the central interest of this paper is the association of work conditions at first interview with functional limitations that occur subsequently, i.e., at later ages, we use age 70 for the predictions (of FL). We chose age 70 to allow for substantial follow-up subsequent to the age at which work effort was reported (in the 50s and early 60s), and, at the same time, to avoid high rates of attrition (loss-to-follow-up and death) at the oldest ages.

**Fig 1:**
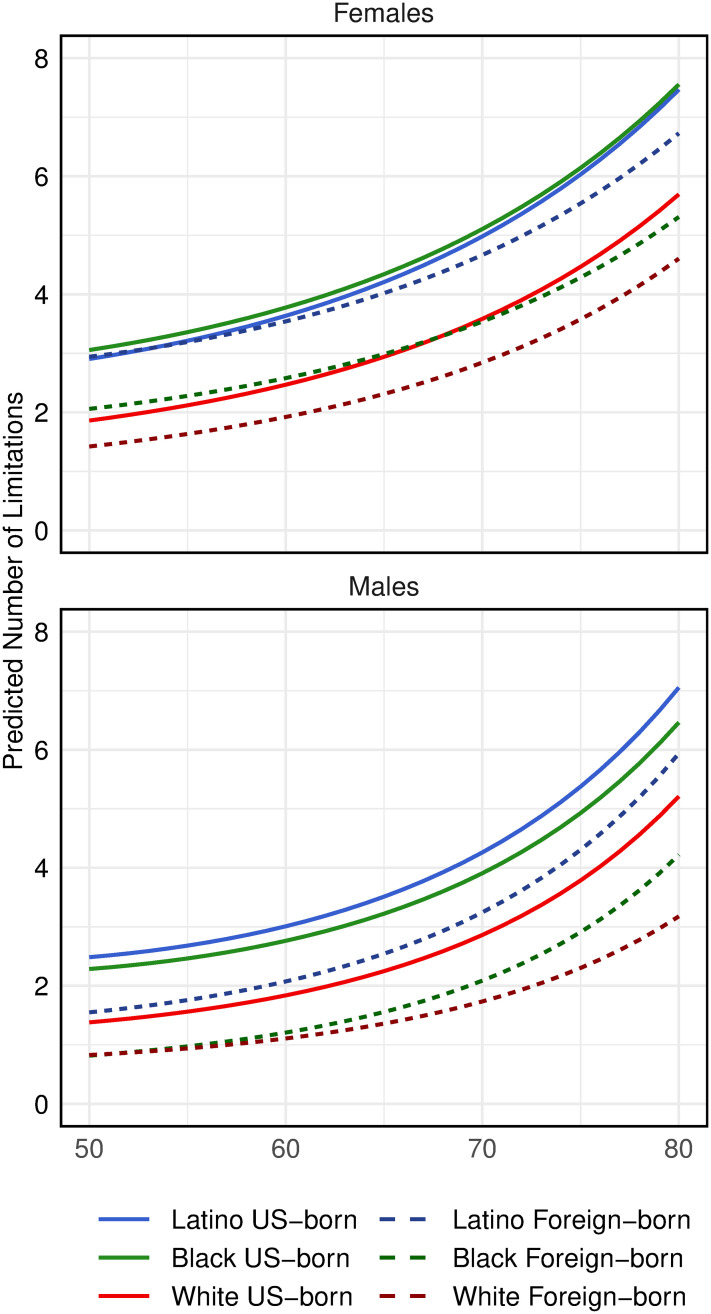
Predicted number of functional limitations by gender, age, and race/ethnicity/nativity. These predictions are based on the HRS 1998–2014 sample, regardless of employment status, which is described in S1 Appendix. Predictions are based on a Poisson model with an individual-level random effect including age (centered on 60 years), age squared, race/ethnicity/nativity (REN), an interaction between age and REN, marital status and wave as predictors.

**Fig 2:**
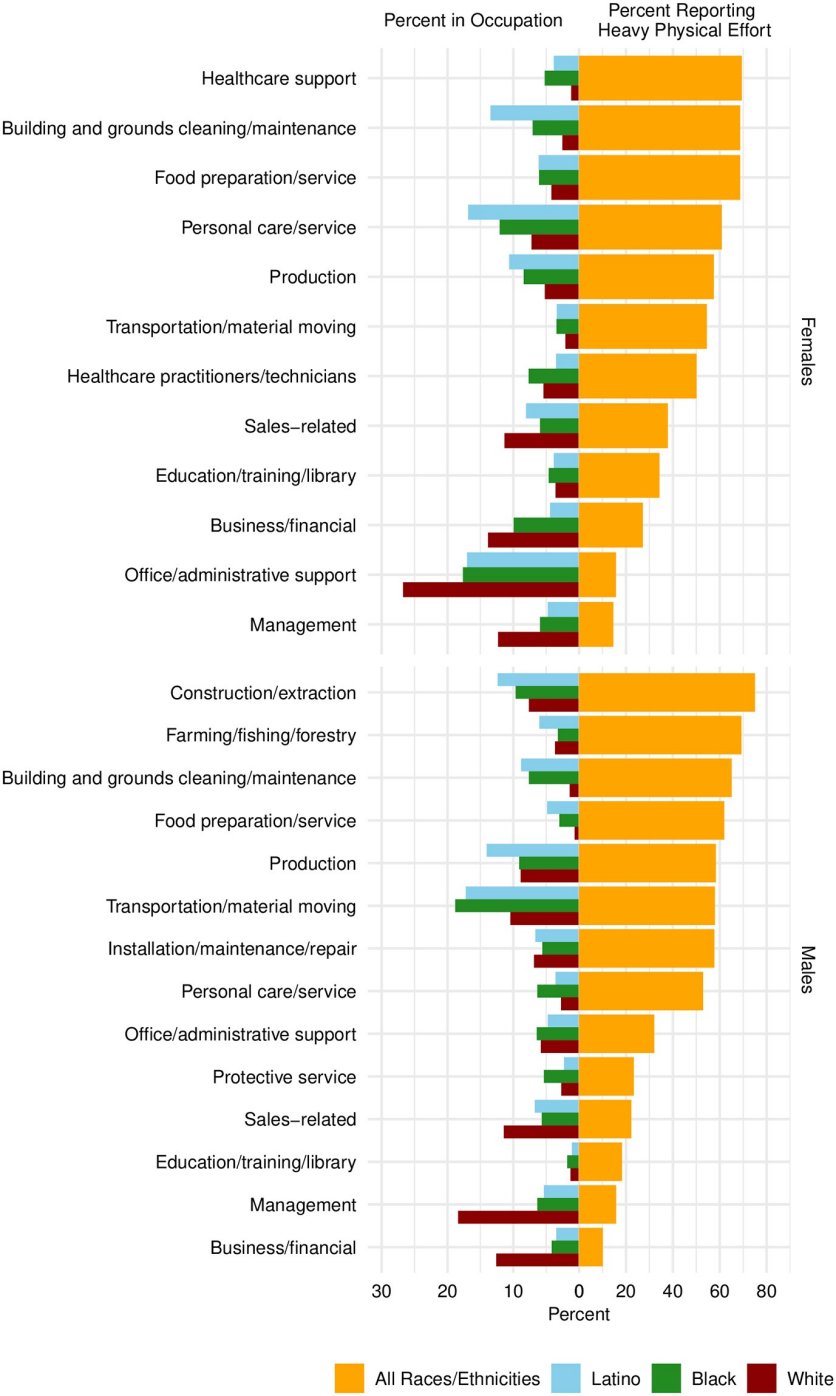
Occupational distribution for the employed sample by race/ethnicity (left) and percent reporting heavy physical effort (right) by occupation, stratified by gender.

All estimates, except for those in Table 1 and Figs 1 and 2, use multiple imputation for missing data. Missing data on time-invariant characteristics were imputed using the MI procedure (Stata version 15.0), producing 10 data sets. To improve the quality of the prediction, the imputation procedure included variables in, and outside of, our analysis (see S2 Appendix). Time-varying characteristics—the number of functional limitations and the health variables—are included in the analysis in their original (non-imputed) form. All analyses are unweighted, though we control for factors associated with sample selection in our multivariate models, an approach that has been shown to produce unbiased and efficient estimates [74, 75]. As noted earlier, all analyses are stratified by gender.

**Table 1:** Summary statistics of the analytic sample of employed HRS respondents: 1998–2014.

Variable	Analytic Sample (N = 17,297)	
	Percent or Mean (SD)	Percent Missing
**Demographic**		
Age (years)	58.2 (7.2)	0
Female	52.4	0
Race/Ethnicity/Nativity		0.05
US-born Latino	4.8	
Foreign-born Latino	7.4	
US-born black	16.7	
Foreign-born black	1.4	
US-born white	66.8	
Foreign-born white	2.8	
Married	69.5	0.1
**Job effort requirements**		
Heavy physical effort	38.3	0
Heavy lifting	22.2	0
**Early life characteristics**		
Father’s years of education	9.7 (4.1)	14.0
Mother’s years of education	10.0 (3.7)	8.0
Respondent’s years of education	12.9 (3.1)	0.4
Lived in rural area	45.1	3.4
Poor health in childhood	5.7	0.2
Childhood SES		0.3
Pretty well off	7.6	
About average	63.9	
Poor	28.5	
Father’s unemployment before age 16		1.2
Never unemployed	73.9	
Unemployed ≥ 3 months	18.1	
Father not around	7.9	
Height (meters)	1.7 (0.1)	0.5
**Adult SES**		
Household income		0
First quartile	16.4	
Second quartile	22.6	
Third quartile	29.3	
Fourth quartile	31.7	
Household wealth		0
First quartile	25.2	
Second quartile	31.2	
Third quartile	25.8	
Fourth quartile	17.8	
**Adult health**		
Number of limitations	1.7 (2.3)	0.2
Ever smoked	58.1	0.3
Ever diagnosed with diabetes	11.4	0.1
Obesity		1.9
Not obese	69.5	
Class 1 (30 ≤ BMI < 35)	20.4	
Class 2 (35 ≤ BMI < 40)	6.8	
Class 3 (40 ≤ BMI < 45)	3.2	

## Results

Table 1 shows summary statistics and the frequency of missing data for the analytic sample. Although sample sizes of most REN subgroups are large, a relatively small proportion of respondents are foreign-born blacks (1.4% or N = 247). The largest groups are US-born whites and blacks and most respondents were married. In more than a third of current jobs, respondents reported that heavy physical effort was required most, almost all or all of the time. Heavy lifting was less common, with about 22 percent of jobs requiring it most, almost all or all of the time. The average educational attainment for respondents’ parents was less than high school (~10 years), but for respondents it was slightly more than the 12 years typically required to complete high school (12.9 years). A sizable minority of respondents reported coming from a poor background or paternal unemployment. Respondents reported an average of 1.7 functional limitations. In terms of health status, 58% had ever smoked, 11% had been diagnosed with diabetes, and about 30% were classified as obese (i.e., BMI ≥30). There is little missing data except for some of the childhood variables, such as paternal and maternal educational attainment and whether the respondent lived in a rural area while growing up.

## Racial/ethnic/nativity differentials in FL

We begin by examining race, ethnic, and nativity differentials in functional limitations. To represent the entire population at these ages in the US, we use the sample of HRS respondents whether or not they were employed at the time of first observation. This sample is described in more detail in S1 Appendix. Using this sample, we estimate a trajectory model of FL (also described in S1 Appendix) with the following basic controls: age, wave, and marital status. Fig 1 displays the mean number of functional limitations by REN and gender from ages 50 through 80 estimated from the model. The graphs reveal the normal rise in FL with increasing age and the relatively higher FL prevalence for women. Throughout this age range, both US-born and foreign-born Latinos have more FL than any group except US-born blacks. For all groups, US-born respondents report more limitations than their foreign-born counterparts, although the difference for Latinas is small for much of the age range. The low FL of foreign-born blacks, relative to all groups except foreign-born whites, is not surprising—earlier research has shown that black immigrants experience considerably lower levels of disability and lower morbidity and mortality than US-born blacks [76, 77]. Estimates for foreign-born blacks are imprecise and should be interpreted with caution due to the relatively small sample size for this group.

For almost all of the individual functional limitations that comprise the FL measure, Latinos report having the particular limitation more frequently than whites, irrespective of nativity and gender. After we adjust for age, marital status, and wave using the multivariate model, either Latinos (mostly US-born Latinos) or US-born blacks have the highest prevalence for any given limitation (see S3 Appendix).

As described above, because HRS collects data on work conditions only during the survey period, our main study is based on a sample of respondents who were employed at or close to their first observation in their 50s or 60s. However, it is quite likely that individuals with higher numbers of FL were especially likely to have exited the labor force before these ages or to have been unemployed at the time of HRS interview, and thereby excluded from our analytic sample of employed persons. If this exclusion occurred more frequently for Latinos than whites, data for the analytic sample would likely show a smaller differential in FL between Latinos and whites than observed in the full sample. Our analysis (S1 Appendix) shows that the Latino disadvantage relative to whites in FL is, indeed, somewhat smaller in the analytic sample. The impact of this reduced disadvantage on our results is considered in the discussion section.

## Racial and ethnic differences in heavy physical effort

After establishing the REN differentials in FL, we describe the distribution of heavy physical effort by race and ethnicity. Fig 2 displays the prevalence of reporting heavy physical effort for the most common occupations alongside the percent in each occupation by race/ethnicity, shown separately for employed men and women. The graph confirms that the highest average physical effort is reported for manual and less skilled occupations, as we would expect. The figure also demonstrates that Latinos are much more likely than whites to hold high work effort occupations and less likely than whites to have jobs requiring lower effort.

To determine whether early life variables and adult SES could account for REN differences in work effort, we also conducted a multivariate analysis in which the outcome variable is work effort (based on separate models for heavy physical effort and heavy lifting) at the respondent’s job (during their 50s or early 60s) and the covariates are age, age squared, REN, early life characteristics, and respondent’s years of education. The results, shown in S4 Appendix, confirm much higher work effort among Latinos and blacks compared with whites. When early life conditions and educational attainment are held constant, the Latino-white gap narrows considerably but the black-white gap remains large.

### Effect of work effort on FL

**Fig 3:**
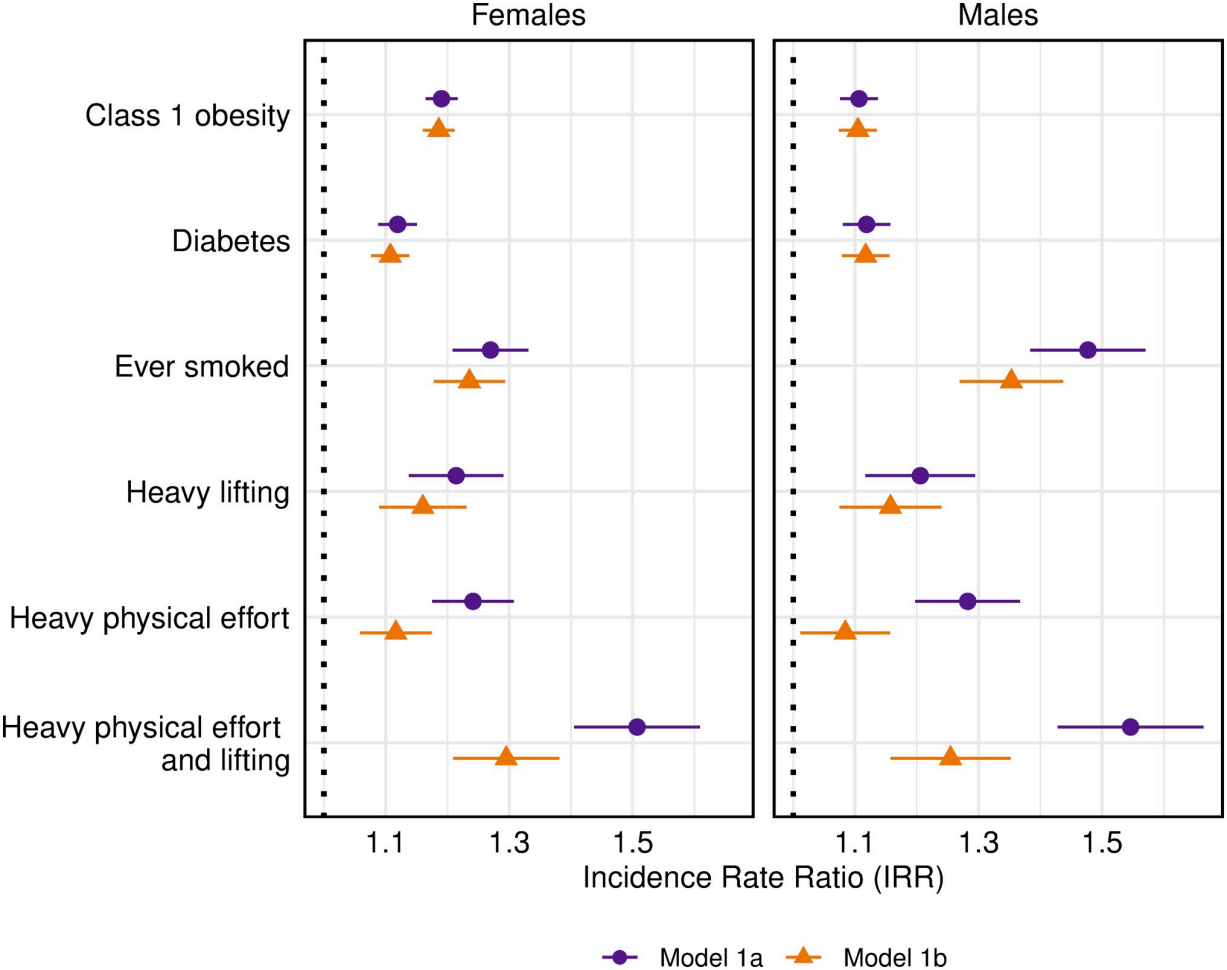
Selected incidence rate ratios (IRRs) and 95% confidence intervals from models predicting number of limitations. Model 1a: age (centered on 60 years), age squared, race/ethnicity/nativity (REN), an interaction between age and REN, marital status, wave, adult health variables and work effort variables. Model 1b: Model 1a plus family financial status from birth to age 16, father’s employment status prior to age 16, mother’s education, father’s education, whether the respondent lived in a rural area most of the time, health status prior to age, height, respondent’s years of education, and measures of adult SES (quartile of household income and quartile of household wealth).

Next we examine the association between physical work effort and functional limitations in the employed sample. We use two trajectory models—Models 1a and 1b, described above. The latter adds early life variables and measures of adult SES to Model 1a. The association between work effort and FL remains statistically significant in Model 1b, as the values in Fig 3 show. To illustrate the relative size of this association, we compare the effect of work effort to those of several health conditions and health behaviors (obesity, diabetes, and smoking), which are known to influence the development of FL; all variables are dichotomous. Fig 3 displays exponentiated coefficients (i.e., the incidence rate ratios or IRRs, which denote the rate of FL for a particular group relative to the complementary reference group) and 95% confidence intervals associated with the work effort measures and with the three health variables.

The IRRs in Fig 3 are shown for: class 1 obesity (vs. non-obese), ever diagnosed with diabetes, ever smoked, heavy lifting, heavy physical effort, and combined heavy physical effort and lifting. Although the IRRs are smaller in Model 1b than Model 1a, as would be expected with the inclusion of childhood variables and adult SES, the effects of heavy physical effort and heavy lifting remain sizeable in Model 1b and on par with the health variables. For example, for both men and women, the IRR for heavy lifting (female IRR = 1.16 (1.09, 1.23); male IRR = 1.16 (1.07, 1.24)) exceeds that for diabetes (female IRR = 1.11 (1.08, 1.14); male IRR = 1.12 (1.08, 1.16)) in Model 1b. The combination of heavy physical effort and heavy lifting (reported by one-sixth of the analytic sample) indicates that those reporting both aspects of work effort have 25–30% more FL than those whose jobs have neither work condition, a relative incidence that exceeds those associated with the health variables, except for ever smoking among men.

### Effect of work effort on racial/ethnic/nativity differences in numbers of limitations

**Fig 4:**
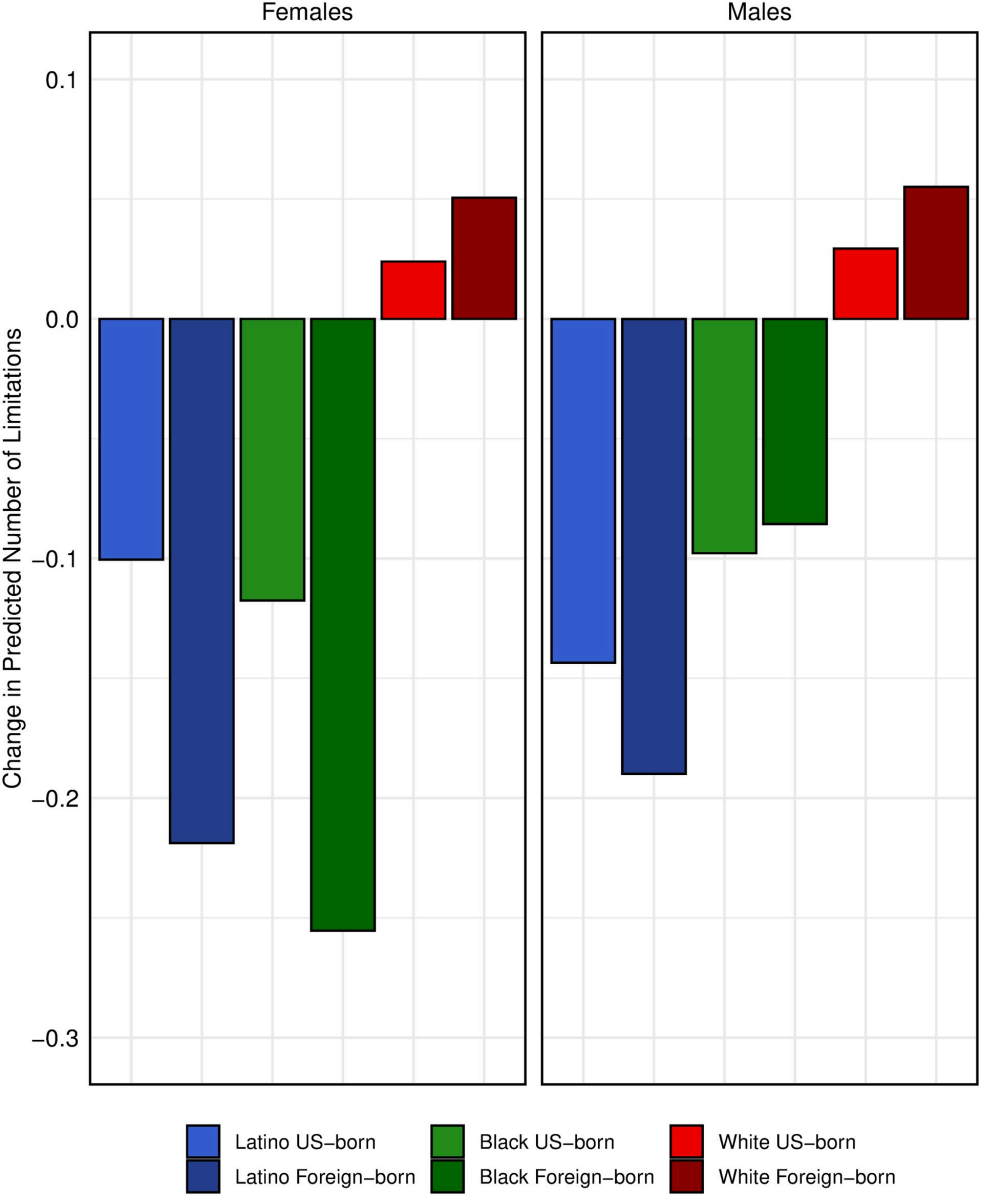
Change in predicted number of limitations at age 70 with inclusion of work effort. The change displayed is the difference in predicted number of limitations at age 70 between Model 2a and Model 2b. Model 2a includes age (centered on 60 years), age squared, race/ethnicity/nativity (REN), an interaction between age and REN, marital status, and wave. Model 2b additionally includes work effort variables.

Above we showed that work effort appears to be strongly associated with functional limitations. Next we explore the extent to which racial/ethnic/nativity differences in work effort can account for racial/ethnic/nativity differences in functional limitations. We do so by assessing how REN differentials in a model change when work effort variables are added to the model. We examine this change in a model with few covariates. Fig 4 (based on the coefficients in the S5 Appendix) presents the *difference* in the predicted number of limitations at age 70 by racial/ethnic/nativity groups between two variants of Model 2: a) without and b) with the work effort variables. These values can be interpreted as the predicted change in the number of functional limitations that a REN group would exhibit if their work effort levels were the same as the entire analytic sample rather than those observed in the particular REN group. For example, Latinos have negative values, holding constant other variables in the model, which means that setting this group’s work effort level to the average for the entire sample results in a smaller predicted number of functional limitations. The reason is that jobs held by Latinos, on average, require higher levels of work effort than those of the sample as a whole. The differences shown in Fig 4 are in the expected direction—a decrease for Latinos and an increase for whites—hence, a reduction in the REN differential—but the magnitudes are modest. For example, including work effort reduces the predicted number of FL by 0.22 for foreign-born Latina women and increases the predicted number of FL by only 0.03 for US-born white women. Moreover, repeating this exercise in models including all predictor variables reduces these differences to virtually zero (estimated coefficients from Model 2c and Model 1b are presented in S5 Appendix). In short, although work effort has a substantial association with FL at older ages, it accounts for almost none of the REN differential once other proximate factors are taken into account.

## Discussion

In this paper, we investigate differences in the prevalence of functional limitations among Latinos compared with whites and blacks, by nativity. We hypothesize that REN differences in physical work conditions contribute to observed REN disparities in physical functional limitations at older ages.

There are several major findings. First, the trajectories of functional limitations at older ages differ substantially by race/ethnicity, nativity, and gender. Latinos—and also blacks—have considerably more FL than whites (Fig 1). Within each racial/ethnic group, functional limitations are less common among the foreign-born than the US-born. However, the number of limitations among foreign-born Latinos still greatly exceeds that among whites. Differences between US-born Latinos and blacks are very small for both men and women. In contrast to Haas and Rohlfsen’s [6] results using earlier waves of HRS and a different parameterization of functional limitations, we find no convergence with age in the number of functional limitations between Latinos and whites. Latinos continue to have a disproportionate share of limitations across older ages.

Second, we find that Latinos, especially immigrants, report more strenuous work conditions than whites, but not blacks (Fig 2 and S4 Appendix). Blacks have more strenuous work conditions than Latinos or whites.

Third, more strenuous work conditions are associated with substantially higher levels of FL during subsequent ages. These results reinforce the importance of investigating the role of work conditions in the association between occupational segregation and health outcomes by social class.

Finally, contrary to expectation, REN differences in later-life work conditions account for a very small portion of Latino-white differences in subsequent FL trajectories. It may be that REN differences are simply not associated with differences in work conditions. However, we believe that this conclusion is premature. An alternative explanation is that data limitations result in an underestimate of the effect of work conditions on FL. The HRS is the best US dataset for this analysis because of its large, nationally-representative longitudinal sample, Latino oversamples, broad range of well-tested measures of physical functioning, and data on respondents’ physical work conditions. However, it has two potentially important limitations. First, as described above, information on respondents’ work conditions is available only during the time period covered by the HRS interviews (ages 50 +). Differentials in work conditions by REN are likely to be larger at younger ages when strength and stamina are greater and workers know less about the labor market—but in current HRS data, we cannot observe this earlier period.

A second limitation is that both work conditions and the FL measure are reported by the respondents themselves, raising the potential for differential reporting bias by REN. This bias would be particularly problematic if Latinos report both poorer working conditions and more frequent functional limitations than whites, even if their actual conditions are identical. However, two other studies using HRS data confirm that Latinos have higher FL than whites. First, using anchoring vignettes to correct potential reporting biases, Dowd and Todd [78] find that Latino-white differentials in reported mobility increase after adjustment by vignette data—indicating that, if anything, Latinos are more likely to underreport FL relative to whites. Second, Haas, Krueger, and Rohlfsen [3] find that in-person tests of grip strength and timed walks in HRS are generally significantly poorer for Latinos than whites, even when compositional differences between the two groups are held constant. In the case of work conditions, estimates from other data that are not self-reported indicate that Latinos, particularly immigrants, hold more physically demanding and hazardous jobs than whites [18, 30]. Moreover, if REN differences in reporting work conditions in HRS were driven primarily by differences in inherent rating scales (i.e., Latinos reporting more effort than whites for similar job conditions), interactions between ethnicity and work effort in our models of functional limitations should be statistically significant, but they are not.

Another important hypothesis about why work conditions in late middle age do not appear to account for Latino-white differentials in FL is differential selection by REN out of employment prior to the first interview. Specifically, as results in the S1 Appendix show, for respondents in their 50s and early 60s, Latinos are less likely than whites to be employed, in part, because they are more likely to have FL at younger ages. Thus, in our analytic sample, the REN differences in FL at the time of first interview are considerably smaller than in the full HRS sample (i.e., a sample that includes both employed and unemployed persons). These earlier work conditions appear to be especially important since they may help to account for why Latinos have higher FL rates and lower labor force participation at the time they are first interviewed.

A related issue is that the association between work conditions and FL may be bidirectional, because FL can limit the types of jobs workers choose or for which they are eligible. Workers who are healthier and stronger may seek out more strenuous jobs if employers offer a wage premium for good physical condition or higher production [79, 80]. This may be particularly true for lower income, more poorly educated workers who have more limited employment opportunities—and thus, this phenomenon may occur more frequently among Latinos than whites. In this analysis, we sought to minimize the potential effect of FL on work conditions by examining the effects of work conditions when respondents were in their fifties and early sixties on *subsequent* FL. Nonetheless, wear and tear, injuries, and disease are often apparent before functional limitations fully develop. Therefore, the types of jobs respondents hold at this stage (and whether they are working at all) may be affected by the same conditions that lead to the development of FL later in life. To the extent that health, strength, and good physical condition lead workers to take jobs with more strenuous work conditions, the association between strenuous work conditions and higher FL would be *weaker* than otherwise.

Despite limitations of the data and the small role of work conditions in accounting for REN differentials in functional limitations in this analysis, our results highlight the importance of considering racial, ethnic, nativity, gender, and social class differentials in physical work conditions in studies of the social determinants of health. During the past 50 years, automation and occupational safety regulations have dramatically reduced heavy physical labor and the risk of occupational injury and death for the US workforce. These changes and others have led researchers to focus primarily on the psychosocial aspects of work. Differentials in psychosocial work conditions by REN are likely to contribute to poorer health for disadvantaged groups at older ages. However, our results and those of other studies show that disadvantaged workers are still much more likely than others to do strenuous physical work on the job. Furthermore, we find that heavier physical work can lead to poorer physical functioning in subsequent years.

If these associations are replicated with data containing more complete work histories, the public health and social implications of these findings are important. The results suggest that investments in improving work conditions at physically strenuous jobs (which are often also low wage and low status jobs) could yield substantial benefits in lowering disability-associated costs—both financial and social—at older ages. Aside from their effects on individuals and families, functional limitations and associated disability increase public expenditures on social programs (e.g., including Medicare, Medicaid, and Supplemental Security Income or SSI), which account for a sizeable portion of governmental budgets, at the national and state levels. They also reduce the productivity of the labor force. Investments in occupational health and policy interventions focused on risk mitigation and improvement of work conditions in physically strenuous work environments may thus be important for reducing the prevalence of functional limitations at older ages and may also yield important benefits for the economy as well. Future research may show that these investments would also reduce race, ethnic, and nativity disparities in disability-related health conditions.

## Supporting information

S1 Appendix Selection into the employment at the time of first observation. (DOCX)

S2 Appendix Description of multiple imputation. (DOCX)

S3 Appendix Predicted probability of individual functional limitations at age 70 adjusting for demographic characteristics. (DOCX)

S4 Appendix Selection of employed individuals into jobs with high levels of work effort. (DOCX)

S5 Appendix Coefficients from random intercept poisson models predicting number of limitations. (DOCX)
