# Supplementary material for: Trajectories of physical functioning among older adults in the US by race, ethnicity and nativity: Examining the role of working conditions
Source: PLoS One. 2021 Mar 17;16(3):e0247804. doi: 10.1371/journal.pone.0247804 (PMC7968635; doi:10.1371/journal.pone.0247804)
Supplement: S1 Appendix — (DOCX) [file pone.0247804.s001.docx]

**S1 Appendix. Selection into employment at the time of first observation**

Our analysis is based on a sample of respondents from the Health and Retirement Survey (HRS) who were between 51 and 66 at their first interview. Because HRS essentially collects information on employment and physical work effort only for the period of the survey itself (1992 to 2014^[[1]](#footnote-1)^), our analytic sample represents only workers who were still employed in their 50s and early 60s. Those who were previously employed but were retired, disabled, or not employed for other reasons at their first HRS interview are excluded. This limitation could affect the results. If workers with jobs requiring heavier work effort are more likely to develop functional limitations, they may retire, go on disability, or be unable to work at earlier ages and be excluded from our sample (for lack of information on jobs and work effort prior to their first HRS interview). For example, Latinos are more likely to work at physically strenuous jobs than white workers. If Latino workers leave the workforce earlier because they are more likely to develop functional limitations, we might incorrectly correctly conclude from our analytic sample that the association between physically strenuous work and higher levels of functional limitations at older ages is smaller than it is in reality for Latinos compared to whites.

To assess the likely magnitude of this problem, we first compare the means or frequency distributions of all the demographic and socioeconomic characteristics of the full HRS sample with those in our analytic sample in Table S1.1. Note that the “full” HRS sample in Table S1.1 was constructed using the procedures and exclusions described in the main text for the analytic sample with the exception that the full sample includes respondents who were *not* working, as well as those who were, at the time of first observation. It also includes a relatively small number of respondents who were working at the time of initial observation but did not report work effort. The full sample includes 159,293 observations of 30,447 respondents (13,215 men and 17,232 women), with an average of 5.3 observations per respondent. Respondent ages range from 50 to 105. The statistics shown in Table S1.1 were calculated from *non*-imputed data (see the Analytic Strategy section in the main text for description of the imputation). The comparison shows that the analytic sample is about five years younger, includes more men, has higher SES, and has fewer limitations. The REN distribution for both samples is quite similar.

Next, we use multivariate modeling to examine selection from the full sample into the analytic sample. Predictor variables in the model include age, age squared, a set of REN dummy variables (Latino US-born, Latino foreign-born, Black US-born, Black foreign-born, white US-born and white foreign-born), the number of FL, and interactions between the number of FL and the REN dummies. The results (in Table S1.2) from logistic models predicting which respondents in the full sample are included in the analytic sample include two important findings. First, coefficients on the REN variables reveal that, for those who report zero functional limitations, foreign-born Latino men and women and US-born Latino men are significantly more likely than US-born whites to be excluded from the analytic sample (i.e., to be not working). Second, coefficients on the main effects for the number of limitations combined with REN interactions indicate that, for each racial/ethnic/nativity group, the more FL that respondents have, the more likely they are to be excluded from the analytic sample (i.e., the less likely they are to be working). The effect of additional limitations is especially large for US-born Latinas (and US-born black men and women).

**Table S1.1: Summary statistics for full HRS sample and analytic sample at first observation**

|  | **Full HRS Sample**  **(N = 30,447)** | | **Analytic Sample**  **(N = 17,297)** | |
| --- | --- | --- | --- | --- |
| **Variable** | **Mean (SD) or Percent** | **Percent Missing** | **Mean (SD) or Percent** | **Percent Missing** |
| **Demographic** |  |  |  |  |
| Age (years) | 63.0 (11.1) | 0% | 58.2 (7.2) | 0% |
| Female | 56.6% | 0% | 52.4% | 0% |
| Race/Ethnicity/Nativity |  | 0.05% |  | 0.05% |
| US-born Latino | 4.8% |  | 4.8% |  |
| Foreign-born Latino | 7.0% |  | 7.4% |  |
| US-born black | 17.1% |  | 16.7% |  |
| Foreign-born black | 1.2% |  | 1.4% |  |
| US-born white | 66.7% |  | 66.8% |  |
| Foreign-born white | 3.2% |  | 2.8% |  |
| Married | 63.6% | 0.1% | 69.5% | 0.1% |
| **Early life characteristics** |  |  |  |  |
| Father’s years of education | 9.2 (3.8) | 16.4% | 9.7 (4.1) | 14.0% |
| Mother’s years of education | 9.4 (3.6) | 10.6% | 10.0 (3.7) | 8.0% |
| Respondent’s years of education | 12.2 (3.4) | 0.3% | 12.9 (3.1) | 0.4% |
| Lived in rural area | 45.0% | 8.7% | 45.1% | 3.4% |
| Poor health in childhood | 6.8% | 0.7% | 5.7% | 0.2% |
| Childhood SES |  | 0.4% |  | 0.3% |
| Pretty well off | 7.0% |  | 7.6% |  |
| About average | 61.6% |  | 63.9% |  |
| Poor | 31.4% |  | 28.5% |  |
| Father’s unemployment before age 16 |  | 1.7% |  | 1.2% |
| Never unemployed | 71.5% |  | 73.9% |  |
| Unemployed ≥ 3 months | 19.5% |  | 18.1% |  |
| Father not around | 8.9% |  | 7.9% |  |
| Height (meters) | 1.7 (0.1) | 0.6% | 1.7 (0.1) | 0.5% |
| **Adult SES** |  |  |  |  |
| Household income |  | 0% |  | 0% |
| First quartile | 31.2% |  | 16.4% |  |
| Second quartile | 24.7% |  | 22.6% |  |
| Third quartile | 23.1% |  | 29.3% |  |
| Fourth quartile | 22.1% |  | 31.7% |  |
| Household wealth |  | 0% |  | 0% |
| First quartile | 29.6% |  | 25.2% |  |
| Second quartile | 30.9% |  | 31.2% |  |
| Third quartile | 24.1% |  | 25.8% |  |
| Fourth quartile | 15.4% |  | 17.8% |  |
| **Adult health** |  |  |  |  |
| Number of limitations | 2.7 (3.1) | 0.7% | 1.7 (2.3) | 0.2% |
| Ever smoked | 58.1% | 0.5% | 58.1% | 0.3% |
| Ever diagnosed with diabetes | 14.1% | 0.1% | 11.4% | 0.1% |
| Obesity |  | 1.9% |  | 1.9% |
| Not obese | 72.4% |  | 69.5% |  |
| Class 1 (30 ≤ BMI < 35) | 18.1% |  | 20.4% |  |
| Class 2 (35 ≤ BMI < 40) | 6.1% |  | 6.8% |  |
| Class 3 (40 ≤ BMI < 45) | 3.4% |  | 3.2% |  |
| Note: Means and percentages are defined for respondents not missing the variable. These statistics refer to the first observation of a respondent between 1998-2014. | | | | |

**Table S1.2: Coefficients from logistic regression models predicting inclusion in the analytic sample**

|  | Females | Males |
| --- | --- | --- |
| Age (centered at age 60) | -0.107*** | -0.090*** |
| Age squared | -0.003*** | -0.003*** |
| Race/Nativity  (ref=US-born white) |  |  |
| Latino US-born | -0.119 | -0.331* |
| Latino foreign-born | -0.566*** | -0.283* |
| Black US-born | 0.196* | -0.312*** |
| Black foreign-born | 0.114 | -0.028 |
| White foreign-born | -0.278^†^ | 0.149 |
| Number of limitations | -0.210*** | -0.255*** |
| Number of Limitations*Race/Nativity |  |  |
| Number of Limitations*Latino US-born | -0.062* | -0.017 |
| Number of Limitations*Latino foreign-born | 0.013 | -0.012 |
| Number of Limitations*Black US-born | -0.083*** | -0.079*** |
| Number of Limitations*Black foreign-born | -0.0002 | -0.102 |
| Number of Limitations*White foreign-born | 0.0004 | 0.015 |
| Constant | 1.374*** | 1.802*** |
| Number of respondents | 17,107 | 13,118 |
| Note: Results pooled from 10 imputations. ^†^ p < 0.10, ^*^ p < 0.05, ^**^ p < 0.01, ^***^ p < 0.001  The numbers of respondents are slightly smaller than those reported in Table S1.1 because some individuals were missing number of limitations at their first observation and are thus not included in this analysis though they go on to report limitations in subsequent waves. | | |

The main consequence of this selection process is that the difference in the number of limitations between the full sample and the analytic sample is greater for Latinos than for whites. This is evidenced by the predicted numbers of FL by REN in each sample, obtained from two Poisson models and shown in Table S1.3. US-born Latinos in the analytic sample have between 0.6 (women) and 0.8 (men) fewer limitations than those in the full sample; the corresponding differences for US-born whites are 0.2 (women) and 0.3 (men) fewer limitations. The net impact is that the Latino-white difference in FL at age 60 is notably smaller in the analytic sample than in the full sample (coefficients from these models are shown in Table S1.4) Thus, although Latinos continue to have more limitations than whites in the analytic sample, our estimates of the impact of work effort are based on a sample with smaller differentials than what we would likely find if we were able to assess past work effort for all respondents and thus did not need to restrict our sample to those still working in their fifties and early sixties.

**Table S1.3: Comparison of predicted number of limitations at age 60 between the full and the analytic sample**

|  | Females | | Males | |
| --- | --- | --- | --- | --- |
|  | Full Sample  (N = 17,028) | Analytic Sample  (N = 8,976) | Full Sample  (N = 13,090) | Analytic Sample  (8,165) |
| Latino US-born | 3.65 | 3.01 | 3.03 | 2.26 |
| Latino Foreign | 3.58 | 3.08 | 2.05 | 1.63 |
| Black US-born | 3.78 | 3.10 | 2.76 | 1.78 |
| Black Foreign | 2.61 | 2.39 | 1.23 | 0.85 |
| White US-born | 2.47 | 2.24 | 1.84 | 1.54 |
| White Foreign | 1.89 | 1.70 | 1.10 | 0.90 |
| Note: Predictions are from the models displayed in Table S1.4. | | | | |

**Table S1.4 Coefficients from random intercept Poisson models predicting number of limitations in full and analytic samples**

|  | Females | | Males | |
| --- | --- | --- | --- | --- |
|  | Full Sample | Analytic Sample | Full Sample | Analytic Sample |
| Age (centered at age 60) | 0.032^***^ | 0.041^***^ | 0.036^***^ | 0.051^***^ |
| Age squared | 0.0005^***^ | 0.0005^***^ | 0.001^***^ | 0.001^***^ |
| Race/Nativity  (ref=US-born white) |  |  |  |  |
| Latino US-born | 0.390^***^ | 0.297^***^ | 0.502^***^ | 0.384^***^ |
| Latino foreign-born | 0.370^***^ | 0.319^***^ | 0.110^*^ | 0.057 |
| Black US-born | 0.426^***^ | 0.325^***^ | 0.407^***^ | 0.143^**^ |
| Black foreign-born | 0.053 | 0.066 | -0.403^***^ | -0.591^***^ |
| White foreign-born | -0.265^***^ | -0.275^**^ | -0.513^***^ | -0.540^***^ |
| Age*Race/Nativity |  |  |  |  |
| Age*Latino US-born | -0.006^**^ | 0.006^†^ | -0.010^***^ | -0.001 |
| Age*Latino foreign-born | -0.010^***^ | -0.007^*^ | 0.0004 | 0.006^†^ |
| Age*Black US-born | -0.007^***^ | 0.001 | -0.010^***^ | -0.0002 |
| Age*Black foreign-born | -0.007^†^ | -0.011^†^ | 0.011 | -0.003 |
| Age*White foreign-born | 0.002 | -0.007^†^ | 0.001 | 0.002 |
| Wave | -0.009^***^ | -0.019^***^ | 0.001 | -0.003 |
| Married | -0.050^***^ | -0.004 | -0.071^***^ | -0.022 |
| Constant | 0.453^***^ | 0.182^***^ | -0.064^**^ | -0.351^***^ |
| Number of observations | 89,890 | 49,431 | 65,952 | 43,786 |
| Number of respondents | 17,028 | 8,976 | 13,090 | 8,165 |
| Note: Results pooled from 10 imputations. ^†^ p < 0.10, ^*^ p < 0.05, ^**^ p < 0.01, ^***^ p < 0.001  The numbers of respondents are slightly smaller than those reported in Table S1.1 because time-varying characteristics were not imputed and observations missing these characteristics were excluded. | | | | |

1. The survey is ongoing but the latest data available at the time of the analysis were from the 2014 interviews. [↑](#footnote-ref-1)
