# Supplementary material for: Trajectories of physical functioning among older adults in the US by race, ethnicity and nativity: Examining the role of working conditions
Source: PLoS One. 2021 Mar 17;16(3):e0247804. doi: 10.1371/journal.pone.0247804 (PMC7968635; doi:10.1371/journal.pone.0247804)
Supplement: S2 Appendix — (DOCX) [file pone.0247804.s002.docx]

**S2 Appendix. Description of multiple imputation**

**Multiple Imputation**

Missing values on time invariant explanatory variables were imputed using the mi command in Stata 15.0 with the chained option. The chained option provides a way to fill in missing values iteratively via chained equations - a sequence of univariate prediction equations that allows specification of different imputation distributions for different types of variables. The imputation model included several variables in addition to the covariates used in the present analysis. All variables used in the imputation were either fixed over time (e.g., race/ethnicity/nativity, gender, measures from childhood) or measured at the first interview in our observation period (e.g., ever having diabetes). The outcome variable (number of limitations) at the first interview was also included in the imputation model but imputed values for this variable were not used in subsequent analysis. Continuous variables were modeled with OLS regression (e.g., height); logistic regression was used for binary variables (e.g., poor health during childhood); Poisson regression was specified for number of limitations; an ordered logit was used for obesity status; and a multinomial logit model was estimated for the other categorical variables (e.g., childhood SES).

Ten multiple imputation datasets were created. Diagnostics showed that the imputed values preserved the distribution of each imputed variable. The models specified in the analysis (Poisson with a random intercept or logistic) were run on each imputed data set. The estimates were then averaged, while the standard errors were calculated to account for variation within and between imputations. The reported predictions from these models at different ages were obtained as averages across the ten imputations.

**Extreme Values**

Extreme values for height in 18 cases were trimmed (values greater than 2.2m and values less than 1.1m). In addition, values of height from the first interview were assigned to all subsequent waves.
