# Supplementary material for: Trajectories of physical functioning among older adults in the US by race, ethnicity and nativity: Examining the role of working conditions
Source: PLoS One. 2021 Mar 17;16(3):e0247804. doi: 10.1371/journal.pone.0247804 (PMC7968635; doi:10.1371/journal.pone.0247804)
Supplement: S3 Appendix — (DOCX) [file pone.0247804.s003.docx]

**S3 Appendix. Predicted probability of individual functional limitations at age 70 adjusting for demographic characteristics**

|  | Females | | | | | |
| --- | --- | --- | --- | --- | --- | --- |
| Limitation | Latino US-born | Latino Foreign-born | Black US-born | Black Foreign-born | White US-born | White Foreign-born |
| Walking several blocks | 0.38 | 0.37 | 0.44 | 0.31 | 0.33 | 0.28 |
| Walking one block | 0.22 | 0.18 | 0.24 | 0.15 | 0.17 | 0.14 |
| Sitting for two hours | 0.33 | 0.31 | 0.26 | 0.19 | 0.21 | 0.20 |
| Getting up from a chair | 0.49 | 0.45 | 0.51 | 0.39 | 0.41 | 0.32 |
| Climbing several flights of stairs | 0.64 | 0.63 | 0.65 | 0.51 | 0.57 | 0.46 |
| Climbing one flight of stairs | 0.33 | 0.34 | 0.30 | 0.20 | 0.22 | 0.18 |
| Stooping, kneeling, crouching | 0.57 | 0.55 | 0.54 | 0.49 | 0.52 | 0.42 |
| Extending arms above shoulders | 0.25 | 0.24 | 0.26 | 0.20 | 0.17 | 0.16 |
| Pushing/pulling large objects | 0.45 | 0.44 | 0.46 | 0.32 | 0.35 | 0.31 |
| Lifting/carrying at least 10 pounds | 0.40 | 0.42 | 0.43 | 0.41 | 0.30 | 0.31 |
| Picking up a dime | 0.10 | 0.10 | 0.10 | 0.09 | 0.08 | 0.07 |
|  | Males | | | | | |
|  | Latino US-born | Latino Foreign-born | Black US-born | Black Foreign-born | White US-born | White Foreign-born |
| Walking several blocks | 0.34 | 0.28 | 0.33 | 0.25 | 0.27 | 0.18 |
| Walking one block | 0.19 | 0.13 | 0.19 | 0.08 | 0.14 | 0.08 |
| Sitting for two hours | 0.27 | 0.23 | 0.21 | 0.15 | 0.17 | 0.13 |
| Getting up from a chair | 0.44 | 0.35 | 0.39 | 0.26 | 0.35 | 0.25 |
| Climbing several flights of stairs | 0.51 | 0.49 | 0.50 | 0.33 | 0.42 | 0.30 |
| Climbing one flight of stairs | 0.26 | 0.23 | 0.21 | 0.12 | 0.15 | 0.10 |
| Stooping, kneeling, crouching | 0.47 | 0.40 | 0.43 | 0.30 | 0.42 | 0.30 |
| Extending arms above shoulders | 0.22 | 0.17 | 0.21 | 0.13 | 0.15 | 0.09 |
| Pushing/pulling large objects | 0.29 | 0.25 | 0.30 | 0.20 | 0.20 | 0.14 |
| Lifting/carrying at least 10 pounds | 0.23 | 0.20 | 0.25 | 0.21 | 0.15 | 0.11 |
| Picking up a dime | 0.12 | 0.09 | 0.09 | 0.07 | 0.06 | 0.05 |

Note: Results pooled from 10 imputations.

Demographic characteristics include age (centered on 60 years), age squared, marital status, and wave.
