# Supplementary material for: Trajectories of physical functioning among older adults in the US by race, ethnicity and nativity: Examining the role of working conditions
Source: PLoS One. 2021 Mar 17;16(3):e0247804. doi: 10.1371/journal.pone.0247804 (PMC7968635; doi:10.1371/journal.pone.0247804)
Supplement: S4 Appendix — (DOCX) [file pone.0247804.s004.docx]

**S4 Appendix. Selection of employed individuals into jobs with high levels of work effort**

In this appendix, we examine factors related to selection of employed individuals into jobs with high levels of work effort. As described in the main text, social background and educational attainment, as well as gender, are likely to be strongly associated with holding strenuous physical jobs and documenting these differences will aid in interpretation of our primary results. Social background and educational attainment differentials may also account for the differentials in the proportions among REN groups in holding strenuous jobs. We examine both overall selection by REN into these jobs and whether these REN differentials are partly accounted for by social background and educational attainment. Since work effort variables are measured once for each respondent at the beginning of observation, the unit of observation is the respondent. Here, we estimate two logistic models: Model A, a model controlling only for age and REN, and Model B, which adds eight early life variables (including educational attainment).

In Fig. S4, we examine selection into strenuous physical work by REN and gender at the individual level and whether this selection could be accounted for by differentials in early life conditions. To do so, we compare Model A with Model B. The first panel of Fig. S4 shows REN and gender differentials in the predicted probabilities of heavy physical effort and 95% confidence intervals associated with these predictions. Model A shows clearly that Latinos, especially the foreign-born, and blacks report higher average physical effort than whites. Once we add childhood and education variables (Model B), the Latino-white differential is substantially reduced, largely because of the dramatic reduction for Latinos, especially for the foreign-born. In other words, the higher prevalence of heavy physical effort for Latinos appears to be largely due to greater early life disadvantage for Latinos compared to whites. In contrast, for blacks and whites, the predicted probability of heavy physical effort changes relatively little between the two models, suggesting that early life factors are not likely to account for the racial difference. The persistence of significantly higher levels of heavy physical effort for blacks compared to whites, even when early life variables are held constant, is consistent with the literature showing that blacks face much greater hurdles to occupational attainment in the US, including structural and institutional forms of discrimination, even when they come from similar backgrounds as whites [1-2]. Estimates of REN differentials in heavy lifting, shown in the second panel, yield similar conclusions. In particular, the reduction in the predicted probability of heavy lifting in the presence of controls for early childhood variables is far greater for Latinos than for the other groups.

**References**

1. Quillian L, Hexel O, Midtbøen AH. Meta-analysis of field experiments shows no change in racial discrimination in hiring over time. Proc Natl Acad Sci U S A. 2017;10;114(41):10870-5.

2. Pager D, Shepherd H. The sociology of discrimination: Racial discrimination in employment, housing, credit, and consumer markets. Annu Rev.Sociol. 2008;34:181-209.

**Fig S4. Predicted probability of reporting heavy physical effort and heavy lifting at age 60.** Model A: age (centered on 60 years), age squared, and race/ethnicity/nativity (REN). Model B: Model A plus family financial status from birth to age 16, father’s employment status prior to age 16, mother’s education, father’s education, whether the respondent lived in a rural area most of the time, health status prior to age 16, height, and respondent’s years of education. Error bars indicate 95% confidence intervals.
