# Supplementary material for: Trajectories of physical functioning among older adults in the US by race, ethnicity and nativity: Examining the role of working conditions
Source: PLoS One. 2021 Mar 17;16(3):e0247804. doi: 10.1371/journal.pone.0247804 (PMC7968635; doi:10.1371/journal.pone.0247804)
Supplement: S5 Appendix — (DOCX) [file pone.0247804.s005.docx]

**S5 Appendix. Coefficients from random intercept Poisson models predicting number of limitations**

|  | Females | | | | Males | | | |
| --- | --- | --- | --- | --- | --- | --- | --- | --- |
|  | Model 2a | Model 2b | Model 2c | Model 1b | Model 2a | Model 2b | Model 2c | Model 1b |
| Age (centered at age 60) | 0.041^***^ | 0.041^***^ | 0.034^***^ | 0.034^***^ | 0.051^***^ | 0.051^***^ | 0.041^***^ | 0.041^***^ |
| Age squared | 0.0005^***^ | 0.0005^***^ | 0.001^***^ | 0.001^***^ | 0.001^***^ | 0.001^***^ | 0.001^***^ | 0.001^***^ |
| Race/Nativity  (ref=US-born white) |  |  |  |  |  |  |  |  |
| Latino US-born | 0.297^***^ | 0.270^***^ | 0.027 | 0.032 | 0.384^***^ | 0.338^***^ | 0.031 | 0.034 |
| Latino foreign-born | 0.319^***^ | 0.261^***^ | -0.150^**^ | -0.141^*^ | 0.057 | -0.020 | -0.520^***^ | -0.509^***^ |
| Black US-born | 0.325^***^ | 0.293^***^ | 0.094^**^ | 0.086^**^ | 0.143^**^ | 0.101^*^ | -0.114^*^ | -0.116^*^ |
| Black foreign-born | 0.066 | -0.019 | -0.027 | -0.065 | -0.591^***^ | -0.661^***^ | -0.690^***^ | -0.709^***^ |
| White foreign-born | -0.275^**^ | -0.260^***^ | -0.209^**^ | -0.202^**^ | -0.540^***^ | -0.521^***^ | -0.432^***^ | -0.428^***^ |
| Age*Race/Nativity |  |  |  |  |  |  |  |  |
| Age*Latino US-born | 0.006^†^ | 0.006^†^ | 0.005^†^ | 0.005^†^ | -0.001 | -0.001 | -0.001 | -0.001 |
| Age*Latino foreign-born | -0.007^*^ | -0.006^*^ | -0.007^*^ | -0.007^*^ | 0.006^†^ | 0.006^†^ | 0.006^†^ | 0.006^†^ |
| Age*Black US-born | 0.001 | 0.001 | 0.001 | 0.001 | -0.0002 | -0.0002 | -0.001 | -0.001 |
| Age*Black foreign-born | -0.011^†^ | -0.011^†^ | -0.011^†^ | -0.011^†^ | -0.003 | -0.004 | -0.003 | -0.003 |
| Age*White foreign-born | -0.007^†^ | -0.007^†^ | -0.006^†^ | -0.006^†^ | 0.002 | 0.002 | 0.001 | 0.001 |
| Wave | -0.019^***^ | -0.018^***^ | -0.008^***^ | -0.008^**^ | -0.003 | -0.003 | 0.014^***^ | 0.013^***^ |
| Married | -0.004 | -0.003 | 0.034^**^ | 0.034^**^ | -0.022 | -0.018 | 0.013 | 0.013 |
| Poor health in childhood |  |  | 0.422^***^ | 0.417^***^ |  |  | 0.293^***^ | 0.293^***^ |
| SES in childhood  (ref = pretty well off) |  |  |  |  |  |  |  |  |
| About average |  |  | 0.022 | 0.032 |  |  | -0.018 | -0.016 |
| Poor |  |  | 0.096^†^ | 0.101^†^ |  |  | 0.031 | 0.035 |
| Lived in rural area |  |  | 0.023 | 0.002 |  |  | 0.061^†^ | 0.051^†^ |
| Father’s unemployment (ref = ≥ 3 months) |  |  |  |  |  |  |  |  |
| Never unemployed |  |  | -0.128^***^ | -0.123^***^ |  |  | -0.170^***^ | -0.163^***^ |
| Father not around |  |  | -0.034 | -0.031 |  |  | -0.015 | -0.015 |
| Mother’s education |  |  | -0.002 | -0.002 |  |  | -0.001 | 0.0001 |
| Father’s education |  |  | -0.017^***^ | -0.017^***^ |  |  | -0.021^***^ | -0.021^***^ |
| Height (meters) |  |  | 0.470^**^ | 0.492^**^ |  |  | 0.311 | 0.332 |
| Respondent’s education |  |  | -0.059^***^ | -0.053^***^ |  |  | -0.065^***^ | -0.057^***^ |
| Income (ref = 1^st^ quartile) |  |  |  |  |  |  |  |  |
| 2^nd^ Quartile |  |  | -0.043^***^ | -0.043^***^ |  |  | -0.042^**^ | -0.042^**^ |
| 3^rd^ Quartile |  |  | -0.078^***^ | -0.077^***^ |  |  | -0.094^***^ | -0.093^***^ |
| 4^th^ Quartile |  |  | -0.115^***^ | -0.112^***^ |  |  | -0.135^***^ | -0.132^***^ |
| Wealth (ref = 1^st^ quartile) |  |  |  |  |  |  |  |  |
| 2^nd^ Quartile |  |  | -0.048^***^ | -0.047^***^ |  |  | -0.089^***^ | -0.089^***^ |
| 3^rd^ Quartile |  |  | -0.095^***^ | -0.093^***^ |  |  | -0.130^***^ | -0.129^***^ |
| 4^th^ Quartile |  |  | -0.159^***^ | -0.156^***^ |  |  | -0.169^***^ | -0.166^***^ |
| Obesity (ref = not obese) |  |  |  |  |  |  |  |  |
| Obesity class 1 |  |  | 0.170^***^ | 0.171^***^ |  |  | 0.099^***^ | 0.100^***^ |
| Obesity class 2 |  |  | 0.325^***^ | 0.325^***^ |  |  | 0.283^***^ | 0.286^***^ |
| Obesity class 3 |  |  | 0.424^***^ | 0.424^***^ |  |  | 0.416^***^ | 0.419^***^ |
| Diabetes |  |  | 0.102^***^ | 0.102^***^ |  |  | 0.110^***^ | 0.111^***^ |
| Ever smoked |  |  | 0.216^***^ | 0.212^***^ |  |  | 0.330^***^ | 0.302^***^ |
| Job Characteristics |  |  |  |  |  |  |  |  |
| Heavy physical effort |  | 0.227^***^ |  | 0.110^***^ |  | 0.257^***^ |  | 0.081^*^ |
| Heavy lifting |  | 0.212^***^ |  | 0.148^***^ |  | 0.199^***^ |  | 0.146^***^ |
| Constant | 0.182^***^ | 0.069^***^ | 0.285^***^ | 0.203^**^ | -0.351^***^ | -0.491^***^ | -0.218^***^ | -0.302^***^ |
| Number of observations | 49,431 | 49,431 | 49,431 | 49,431 | 43,786 | 43,786 | 43,786 | 43,786 |
| Numbers of respondents | 8,976 | 8,976 | 8,976 | 8,976 | 8,165 | 8,165 | 8,165 | 8,165 |
| ^†^ p < 0.10, ^*^ p < 0.05, ^**^ p < 0.01, ^***^ p < 0.001  Note: Results pooled from 10 imputations. The numbers of respondents are slightly smaller than those reported in Table 1 because time-varying characteristics were not imputed and observations missing these characteristics were excluded. | | | | | | | | |
